# A review of sample sizes for UK pilot and feasibility studies on the ISRCTN registry from 2013 to 2020

**DOI:** 10.1186/s40814-023-01416-w

**Published:** 2023-11-21

**Authors:** Nikki Totton, Jinfeng Lin, Steven Julious, Mahima Chowdhury, Andrew Brand

**Affiliations:** 1 School of Health and Related Research, University of Sheffield, Regent Court, 30 Regent Street, Sheffield, S1 4DA UK; 2 Medicine, Dentistry and Health, University of Sheffield, Sheffield, UK; 3 North Wales Organisation for Randomised Trials in Health & Social Care, Bangor University, Bangor, Wales

**Keywords:** Pilot, Feasibility, Sample size, Review, ISRCTN

## Abstract

**Background:**

Pilot and feasibility studies provide information to be used when planning a full trial. A sufficient sample size within the pilot/feasibility study is required so this information can be extracted with suitable precision. This work builds upon previous reviews of pilot and feasibility studies to evaluate whether the target sample size aligns with recent recommendations and whether these targets are being reached.

**Methods:**

A review of the ISRCTN registry was completed using the keywords “pilot” and “feasibility”. The inclusion criteria were UK-based randomised interventional trials that started between 2013 (end of the previous review) and 2020. Target sample size, actual sample size and key design characteristics were extracted. Descriptive statistics were used to present sample sizes overall and by key characteristics.

**Results:**

In total, 761 studies were included in the review of which 448 (59%) were labelled feasibility studies, 244 (32%) pilot studies and 69 (9%) described as both pilot and feasibility studies. Over all included pilot and feasibility studies (*n* = 761), the median target sample size was 30 (IQR 20–50). This was consistent when split by those labelled as a pilot or feasibility study. Slightly larger sample sizes (median = 33, IQR 20–50) were shown for those labelled both pilot and feasibility (*n* = 69). Studies with a continuous outcome (*n* = 592) had a median target sample size of 30 (IQR 20–43) whereas, in line with recommendations, this was larger for those with binary outcomes (median = 50, IQR 25–81, *n* = 97). There was no descriptive difference in the target sample size based on funder type. In studies where the achieved sample size was available (*n* = 301), 173 (57%) did not reach their sample size target; however, the median difference between the target and actual sample sizes was small at just minus four participants (IQR −25–0).

**Conclusions:**

Target sample sizes for pilot and feasibility studies have remained constant since the last review in 2013. Most studies in the review satisfy the earlier and more lenient recommendations however do not satisfy the most recent largest recommendation. Additionally, most studies did not reach their target sample size meaning the information collected may not be sufficient to estimate the required parameters for future definitive randomised controlled trials.

**Supplementary Information:**

The online version contains supplementary material available at 10.1186/s40814-023-01416-w.

## Background

Pilot and feasibility studies (PAFS) are preliminary studies that are used to assess the utility of undertaking a future definitive randomised controlled trial (RCT) which will test the effect of an intervention [1]. PAFS does this by completing a small-scale study where the methodological approach can be trialled and evaluated [2]. Key aspects to evaluate include: assessing which interventions have the best potential to be successful [3], identifying any potential issues with the study design [4] and gathering information to inform the sample size calculation of the future definitive RCT [5].

The definitions of pilot and feasibility studies were inconsistent in the literature, but following consensus exercises by Eldridge et al. [1], it was concluded that a feasibility study is one where the main aim is to assess whether a definitive RCT is plausible. A pilot study is a specific type of feasibility study which uses a small-scale version of the definitive RCT to assess these questions of plausibility. Eldridge and colleagues are clear that these terms are not mutually exclusive [1]; hence, both are included in this review.

It has been suggested that PAFS could be an essential prerequisite to completing a definitive study [5], and they are believed to encourage higher-quality definitive RCTs [6]. Cooper et al. [7] state therefore that the sample size chosen for these preliminary studies must be suitable to achieve the main objectives.

There is no one guideline for choosing an appropriate size for a PAFS [8] with different recommendations provided by a number of authors. Although some authors suggest choosing a sample size based on the sample size estimate of the definitive RCT [9], some have provided flat rates as a rule of thumb. These rules of thumb are Julious [10] who suggests 12 participants per arm and Kieser et al. [11] suggesting between 10 and 20 per arm. These values have increased in more recent work by Sim and Lewis [12] which has suggested 55 participants in total considering a traditional two-arm study and Teare et al. [13] stating 35 patients per arm for continuous outcomes and 60 per arm for binary outcomes will ensure suitable precision. All of these recommendations are based on the precision of the key estimates which are required within a definitive RCT sample size. Aside from Teare et al. [13] who specifically mention both continuous and binary outcomes to estimate the standard deviation or control group rate, respectively, the other recommendations are all based on the standard deviation estimate and therefore are assuming a continuous outcome in the definitive RCT. It is not recommended to include the estimates of treatment effects which are gathered from PAFS as these are known to be prone to bias [14].

Whitehead et al. [7] criticise the rule of thumb approach stating that the size of the full RCT should be taken into account to minimise the sample size across both studies. This varied the rules of thumb depending on the anticipated effect sizes within the full RCT (again assuming a continuous outcome). They recommended that for a 90% powered definitive RCT 10, 15, 25 or 75 patients per arm are required for large (≥ 0.7), medium (0.3–0.69), small (0.1–0.29) and extra small (< 0.1) effect sizes, respectively. Given this variation in recommendations, the presence of a justification for the sample size chosen for PAFS has been identified as a key area for future improvements [15].

A review by Billingham et al. [16] was completed to assess the sample size targets of ongoing PAFS (*n* = 79) in 2013. They found a median sample size target of 30 (IQR 20–45) participants per arm for pilot studies (*n* = 50) and 36 (IQR 25–50) for feasibility studies (*n* = 25), although these had large variations. The authors also noted a slight difference in the median target sample sizes for different endpoints (36 (IQR 25–50) for binary, 30 (IQR 20–50) for continuous). These sample sizes satisfy the earlier recommendations of Julious [10] (*n* = 12), Kieser et al. [11] (*n* = 10–20) and Sim and Lewis [12] (*n* = 55 total) as outlined above. However, those by Teare et al. [13] (*n* = 60 for binary, *n* = 35 for continuous) were not satisfied.

The work by Arain et al. [17] used pilot and feasibility studies in the literature (*n* = 54) to evaluate the sample sizes achieved in these studies, finding a median total number of participants of 62.5 (IQR 31, 189) and 125.5 (36, 1005) for pilot (*n* = 20) and feasibility (*n* = 34) studies, respectively. For mostly two-armed studies, the value for pilot studies is consistent with that found by Billingham et al. [16] as the target sample size, however much higher numbers for feasibility studies. This could be due to a difference in the studies that were included within each review with Arain et al. not limiting their review to only randomised studies. A review of definitive RCTs published in the National Institute for Health Research (NIHR) Journals Library between 1997 and 2020 [18] found that only 63% (245/388) of RCTs recruited to their target sample size with further work suggesting that the difference between target and actual sample sizes in definitive RCTs is increasing over time [19]. As one of the aims of PAFS is to test the potential to recruit participants, there could potentially be more studies that have issues recruiting to target. Therefore, considering both the target and final sample size for PAFS will provide useful information as to whether, not only the target sample size of a study is being set in line with the recommendations outlined above, but also how often this sample size is being achieved.

This review aims to gain an up-to-date overview of the target sample sizes used in PAFS. Of particular interest is whether this has changed since the previous review in 2013 as recommendations in 2012 [12] and 2014 [13] have suggested larger sample sizes for PAFS (28–60 per arm). Additionally, this review aims to assess, where possible, the actual sample size achieved by the pilot/feasibility study and whether this satisfies these targets.

## Methods

The International Standard Randomised Controlled Trial Number (ISRCTN) registration website is used by the International Committee of Medical Journal Editors (ICMJE) to verify public trials and shared in the World Health Organization (WHO) trials search system [20]. Therefore, many researchers register their RCTs on this website providing a rich, freely available, dataset to evaluate the characteristics of PAFS.

The ISRCTN database was searched for the terms “pilot” and “feasibility” on three separate occasions in September 2019 and July 2020 (for use in specific projects) with the final search completed on the 10th March 2022 to ensure data up to the end of 2020 was included. All identified entries were downloaded directly from the webpage, and duplicates were removed between the two downloads before screening took place. Screening and data collection took place independently by a single researcher (MC/JL/NT), and a 25% sample was checked by a second researcher (NT/JL). General characteristics of the studies as well as sample size information were included in the downloaded records, and a full list of the extracted data can be found in Appendix 1.

The inclusion criteria were the following:

• Randomised studies containing at least two treatment arms
• Interventional studies
• Described as either a pilot or feasibility study
• Study that started between 2013 and 2020
• Study completed within the UK

The exclusion criteria were the following:

• A non-parallel groups study including cross-over and factorial designs as these include additional complexity in sample size calculations
• Cluster randomised trials and adaptive designs (for reasons described above)
• Studies in healthy volunteers
• Internal pilots [21] due to the differing sample size considerations

### Analysis

Descriptive statistics on the characteristics of the studies were calculated for the whole dataset as well as split by certain characteristics of interest, namely:

- 1. Whether the study was labelled as a pilot, feasibility or both
- 2. The funder (charity/industry/public/other)
- 3. Endpoint (binary/categorical/continuous/time-to-event)

The categories for these characteristics were decided by MC and NT after the first extraction of data and used consistently thereafter.

Medians and interquartile ranges were used to summarise continuous variables due to the expected skew of the data and frequencies and percentages for categorical variables.

The Preferred Reporting Items for Systematic Reviews and Meta-Analyses (PRISMA) checklist has been followed when reporting the results of this study as appropriate.

## Results

### Screening

**Fig. 1:**
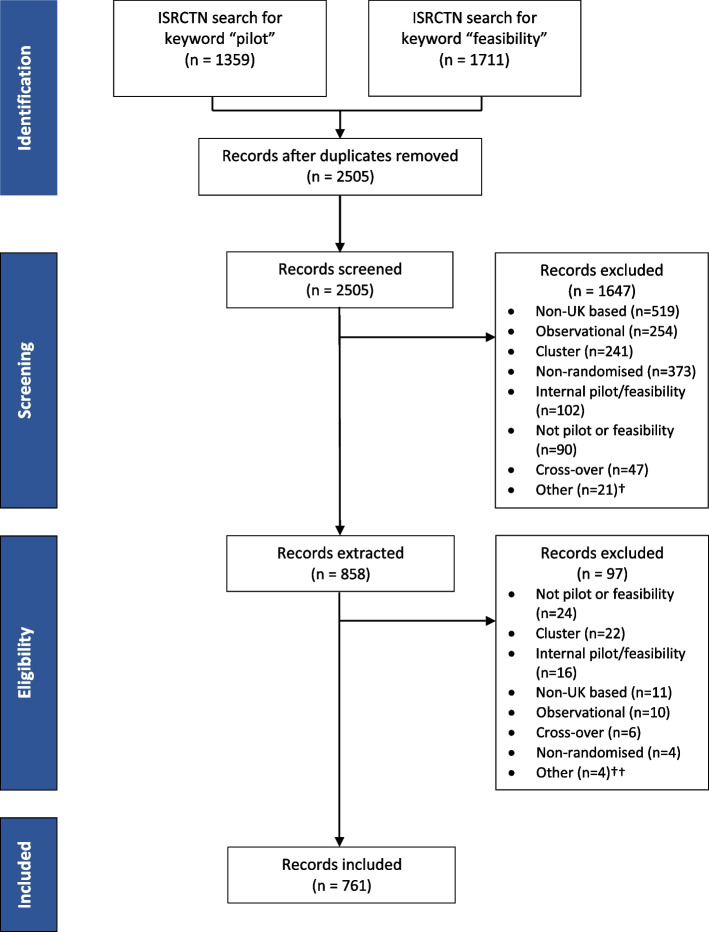
Flow diagram showing the flow of studies between 2013 and 2020 through the review

The search of the ISRCTN database yielded 1711 studies with the search term “feasibility” and 1359 studies with the search term “pilot”. Records for each study were downloaded from the ISRCTN webpage, and after eliminating duplicates and removing any studies not meeting the inclusion criteria, 761 studies went on to be analysed. Of those included, 448 (59%) had been labelled as a feasibility studies, 244 (32%) as a pilot study and 69 (9%) had been described as both a pilot and a feasibility study (Table 1). Figure 1 shows the flow of studies through the review.

**Table 1:** Characteristics of the pilot and feasibility studies included within the review (*n* = 761)

	**Study type**			
	**All**	**Pilot**	**Feasibility**	**Both**
	*N* = 761	*n* = 244	*n* = 448	*n* = 69
**Year study start**	*Frequency (%)*			
2013	97 (13%)	55 (23%)	33 (7%)	9 (13%)
2014	111 (15%)	36 (15%)	69 (15%)	6 (9%)
2015	119 (16%)	44 (18%)	71 (16%)	4 (6%)
2016	101 (13%)	30 (12%)	64 (14%)	7 (10%)
2017	87 (11%)	21 (9%)	55 (12%)	11 (16%)
2018	122 (16%)	26 (11%)	89 (20%)	7 (10%)
2019	73 (10%)	17 (7%)	39 (9%)	17 (25%)
2020	51 (7%)	15 (6%)	28 (6%)	8 (12%)
**Length of study (months)** *Mean (SD)*	32 (19)	29 (19)	33 (18)	35 (20)
**Treatment arms**				
2	680 (89%)	219 (90%)	400 (89%)	61 (88%)
3	64 (8%)	20 (8%)	38 (9%)	6 (9%)
4	15 (2%)	4 (2%)	9 (2%)	2 (3%)
6	2 (< 1%)	1 (< 1%)	1 (< 1%)	0 (0%)
**Primary endpoint**				
Binary	97 (13%)	34 (14%)	54 (12%)	9 (13%)
Categorical	1 (< 1%)	1 (< 1%)	0 (0%)	0 (0%)
Continuous	592 (78%)	191 (78%)	357 (80%)	44 (64%)
Time to event	24 (3%)	6 (3%)	12 (3%)	6 (9%)
Unknown	47 (6%)	12 (5%)	25 (6%)	10 (15%)
**Condition category**				
Mental and behavioural disorders	187 (25%)	66 (27%)	100 (22%)	21 (30%)
Cancer	72 (10%)	13 (5%)	49 (11%)	10 (15%)
Circulatory system	72 (10%)	26 (11%)	40 (9%)	6 (9%)
Musculoskeletal diseases	55 (7%)	12 (5%)	40 (9%)	3 (4%)
Nutritional, metabolic, endocrine	55 (7%)	20 (8%)	32 (7%)	3 (4%)
Nervous system diseases	46 (6%)	12 (5%)	28 (6%)	6 (9%)
Unknown	32 (4%)	14 (6%)	16 (4%)	2 (3%)
Other (groups that represent less than 5%)	242 (32%)	81 (33%)	144 (32%)	19 (26%)
**Study type**				
Treatment	568 (75%)	161 (66%)	354 (79%)	53 (77%)
Quality of life	67 (9%)	27 (11%)	36 (8%)	4 (6%)
Prevention	63 (8%)	27 (11%)	31 (7%)	5 (7%)
Screening	12 (2%)	9 (4%)	3 (1%)	0 (0%)
Diagnostic	8 (1%)	3 (1%)	4 (1%)	1 (1%)
Other	43 (6%)	17 (7%)	20 (5%)	6 (9%)
**Intervention type**				
Behavioural	231 (30%)	61 (25%)	139 (31%)	31 (45%)
Procedure/surgery	64 (8%)	19 (8%)	41 (9%)	4 (6%)
Drug	50 (7%)	21 (9%)	29 (6%)	1 (1%)
Device	43 (6%)	17 (7%)	24 (5%)	2 (3%)
Mixed	24 (3%)	10 (4%)	12 (3%)	2 (3%)
Supplement	18 (2%)	7 (3%)	10 (2%)	1 (1%)
Biological/vaccine	4 (1%)	1 (< 1%)	2 (< 1%)	1 (1%)
Unknown	2 (< 1%)	1 (< 1%)	1 (< 1%)	0 (0%)
Other	325 (43%)	107 (44%)	191 (43%)	27 (39%)
**Funder type**				
Public	557 (73%)	160 (66%)	348 (78%)	49 (71%)
Charity	137 (18%)	49 (20%)	71 (16%)	17 (25%)
Industry	50 (7%)	29 (12%)	20 (5%)	1 (1%)
Other	17 (2%)	6 (3%)	9 (2%)	2 (3%)

### Study characteristics

The characteristics of the studies that were included in the review are shown in Table 1. This shows that study numbers were suitably spread over the years 2013 to 2020. The mean length of all studies was 32 months (standard deviation (SD) = 19) with a slightly longer length for those labelled both a pilot and feasibility (mean = 35, SD = 20) or feasibility alone (mean = 33, SD = 18) than pilot studies (mean = 32, SD = 19). Most studies have two treatment arms (680/761, 89%). The primary endpoint which would be used in a definitive RCT was most commonly a continuous endpoint (592/761, 78%) with binary endpoints only found in 97/761 (13%) studies. These characteristics were consistent across all study types (i.e., pilot, feasibility and both).

The most common condition category for all PAFS was mental and behavioural disorders (187/761, 25%), followed by cancer and circulatory system both with 72/761 (10%) each. The percentages differed slightly depending on the study type with circulatory system (26/244, 11%) being more prominent than cancer (13/244, 5%) in pilot studies but feasibility and both pilot and feasibility studies being the opposite. In line with the common condition categories, behavioural interventions were the most prominent intervention type (231/761, 30%) followed by procedure/surgery representing only 64/761 (8%) of the studies.

The largest source of funding for all studies was public funds (557/761, 73%), and this included government funding programmes such as NIHR and MRC as well as NHS-funded projects. Charity-funded studies were the next most common (137/761, 18%), finally followed by industry-funded studies (50/761, 7%), again with this being consistent across study types.

### Sample sizes

The median sample sizes for all studies and split by the key characteristics of interest are summarised in Table 2. This shows an overall value of 30 (IQR 20–50, *n* = 761) per arm, and this was consistent for those labelled pilot and feasibility studies separately but was slightly higher (median = 33, IQR 20–50, *n* = 69) for those labelled as both a pilot and feasibility study.

**Table 2:** Median sample size per arm overall and split by study type, endpoint and funder

	***N***	**Target sample size**		
		**Median (IQR)**	**Minimum**	**Maximum**
**All studies**	761	30 (20–50)	4	1598
**Study type**				
Pilot	244	30 (20–55)	4	1598
Feasibility	448	30 (21–43)	5	400
Both	69	33 (20–50)	8	125
**Endpoint**				
Binary	97	50 (25–81)	5	400
Categorical	1	200 (200–200)	200	200
Continuous	592	30 (20–43)	4	1598
Time to event	24	39 (30–51)	10	125
Unknown	47	30 (22–41)	10	100
**Funder**				
Charity	137	30 (20–42)	5	1598
Industry	50	30 (20–60)	6	350
Public	557	30 (21–50)	4	650
Other	17	20 (15–25)	5	80

The median sample size per arm was lower for studies using continuous endpoints (30, IQR 20–43, *n* = 592) than studies using binary endpoints (50, IQR 25–81, *n* = 97) but when split by funder all medians were the same at 30 participants per arm, aside from those labelled other which was slightly lower.

Sample size summaries for additional characteristics (condition, study type and intervention type) can be found in Appendix 2. For conditions, this suggests an increased median sample size in infection and infestation studies (50, IQR 35–88, *n* = 24) and neonatal disease (57, IQR 35–87, *n* = 4). Similarly, for study types, there were larger median sample sizes for diagnostic studies (63, IQR 41–79, *n* = 8) and screening studies (60, IQR 40–245, *n* = 12). Assessing intervention type showed only a reduced median sample size for biological/vaccine studies (17, IQR 14–27, *n* = 4).

**Fig. 2:**
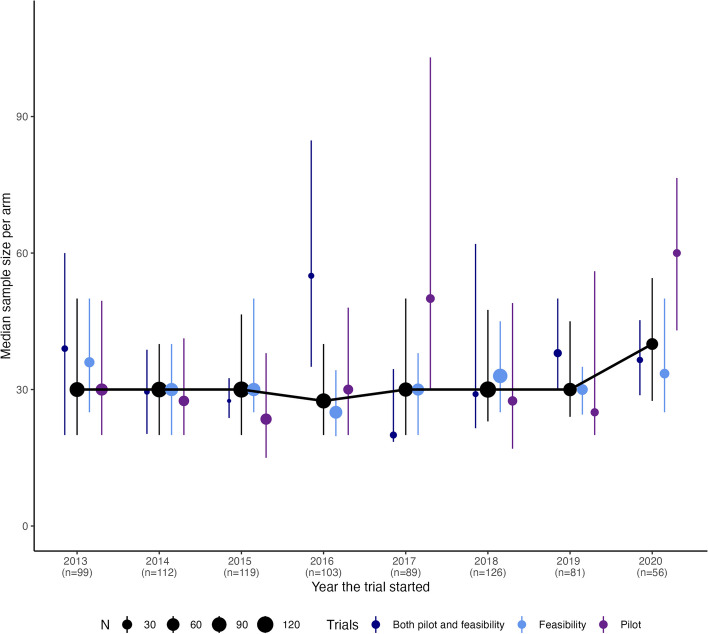
Plot of median and IQR (as error bars) for sample size per arm for studies included within review both over all studies and split by study type

The plot in Fig. 2 shows the sample sizes per arm of studies over time, and the overall plot shows a consistent level between 2013 and 2020 with a slight increase in the final 2 years. However, when looking at this by study type, there is some variation between the years with the majority of the sample size increase in 2020 appearing to be due to pilot studies.

Due to the large maximum value found in the review for the sample size by arm, the twenty largest within the review have been descriptively evaluated further to identify any common characteristics. These had a range of sample sizes per arm from 175 to 1598. The two most common conditions were cancer (3/20, 15%) and urological and genital diseases (3/20, 15%) which differs from the summaries of all PAFS. Seven of the twenty (35%) were studies based on GP practices. The most common study type is treatment (9/20, 45%) as with the characteristics of all PAFS; however, both prevention (5/20, 25%) and screening (4/20, 20%) are more prominent. Finally of note is that 10 of the 20 studies (50%) with the largest sample sizes by arm have binary primary endpoints which does not align with the general characteristics of the review.

The sample size recommendations outlined in the introduction have been summarised in Table 3, along with the number and percentage of studies in the review that satisfy these recommendations. For the smallest recommended sample size (Kieser et al. [11], ≥ 10 per arm), only 11/761 (1%) of the studies do not satisfy these recommendations. However, considering the largest recommendation (Teare et al. [13], continuous outcomes ≥ 35 per arm and binary outcomes ≥ 60 per arm), this increases to 436/761 (57%) that do not satisfy the recommendation. As the recommendations by Whitehead et al. [8] differ from a rule-of-thumb approach for all studies, these have been summarised based on the PAFS that have sample sizes which are appropriate for different effect sizes for the definitive RCT (defined as extra small (< 0.1), small (0.1–0.29), medium (0.3–0.69) and large (≥ 0.7) effect sizes). This shows that 51% (389/761) of the studies could be used to design a definitive RCT with a small effect size and this increases to 72% of studies for a medium effect size.

**Table 3:** Studies in the review that satisfy the different sample size recommendations

**Recommendation**	**Frequency (%)** *N* = 761
Kieser et al. [11] *Continuous outcomes that have* ≥ *10 per arm*	
Satisfied	580 (76%)
Not satisfied	11 (1%)
Not applicable	170 (22%)
Julious [10] *Continuous outcomes that have* ≥ *12 per arm*	
Satisfied	563 (74%)
Not satisfied	28 (4%)
Not applicable	170 (22%)
Sim and Lewis [12] *Continuous outcomes that have* ≥ *28 per arm*	
Satisfied	329 (43%)
Not satisfied	262 (35%)
Not applicable	170 (22%)
Teare et al. [13] *Continuous outcomes that have* ≥ *35 per arm and binary outcomes that have* ≥ *60 per arm*	
Satisfied	251 (33%)
Not satisfied	436 (57%)
Not applicable	74 (10%)
Whitehead et al. [7] *Definitive RCTs with a continuous outcome with a target effect size of:*	
Extra small (< 0.1) (≥ 75 per arm)	52 (7%)
Small (0.1–0.29) (≥ 25 per arm)	389 (51%)
Medium (0.3–0.69) (≥ 15 per arm)	549 (72%)
Large (≥ 0.7) (≥ 10 per arm)	580 (76%)
Not applicable	181 (24%)

### Sample size achieved

For those with final sample size information, 173/301 (57%) did not reach their target total sample size for the feasibility/pilot study; however, 314/615 (51%) of all completed studies did not have this information included on the ISRCTN registry for this to be evaluated (Table 4). The median difference between the target and achieved total sample size was − 4 (IQR − 25 to 0), i.e. the target was missed by four participants with the median percentage of sample size achieved being 93% (IQR 65 to 100%).

**Table 4:** Target and achieved total sample size for all completed studies in the review

	**All completed studies** *N* = 615
**Target sample size achieved?**	**Frequency (%)**
No	173 (28%)
Yes	128 (21%)
Unknown	314 (51%)
	**Median (IQR)**
**Target total sample size**	60 (40, 100)
**Total sample size achieved**	57 (35, 83)
**Difference in target and achieved sample size**	− 4 (− 25, 0)
**Percentage of final sample size achieved**	93% (65%, 100%)

## Discussion

This review of pilot and feasibility studies from 2013 to 2020 found the median sample size per arm across both pilot (30, IQR 20 to 55) and feasibility studies (30, IQR 21 to 43) to be consistent to that found by Billingham et al. [16] for pilot studies (30, IQR 20 to 45) but slightly lower than those labelled feasibility (36, IQR 25 to 50). However, the similar values found suggest this has not changed much over time since their review in 2013.

The value of 30 participants per arm satisfies the recommendations outlined by three of the four rules of thumb previously mentioned. Assessing this further found that for the most lenient (Kieser et al. [11]), which suggests a minimum of 10 participants per arm, was not satisfied by only 11/761 (1%) of studies. Furthermore, over half of the applicable studies are satisfying the recommendations of both Julious [10] and Sim and Lewis [12]. Although the review did find, in line with the recommendations of Teare et al. [13], PAFS with a binary outcome have larger median sample sizes per arm (50, IQR 25 to 81) than those with continuous outcomes (30, IQR 20 to 43), and neither of these values satisfies the recommendations of 60 and 35 per arm, respectively. The values found for continuous outcomes are consistent with the work of Billingham et al. [16]; however, they identified a slightly smaller value for binary outcomes studies (median = 36, IQR 25 to 50).

A study of target effect sizes in definitive RCTs published in the Health Technology Assessment journal found the median target effect size was 0.3 [22]; therefore, based on the sample sizes found in this review and the guidelines by Whitehead et al. [8], 72% (549/761) of the studies had an appropriate target sample size to assist in the design of a definitive RCT consistent with this.

There was no distinction in sample size per arm between the main funder types (charity, industry or public) which all found a median of 30. However, a lower value (median = 20, IQR 15 to 25) was found with those with a funder classed as other. This included investigator-funded and social enterprises; however, there were comparatively very few of these. It was most common for the studies to be publicly funded representing 73% (557/761) of the studies included.

Overall, sample sizes of PAFS found in this review were consistent over the years from 2013 until 2018 with then a potential increase in 2019 and 2020. This finding could be a temporary spike or could represent the start of a gradual increase. Further work could assess whether this trend has continued in years post-2020.

Over half of the studies which we had information did not reach their sample size target (173/301, 57%), this is slightly lower than the 63% (245/388) found by Jacques et al. [18] which considered all definitive RCTs. This is to be expected as these studies are used as a precursor to test the viability of conducting a definitive RCT and inform its design with one of the main elements being tested being whether the study can recruit to target. Understanding any differences in the characteristics of those PAFS that did/did not recruit to target would be an interesting piece of further research. The median difference found between the target and actual sample sizes however was an under-recruitment of just four (IQR − 25 to 0) participants suggesting the studies were not missing their targets by much. This finding is based on about half of the studies that have been completed as only 301/615 (49%) of studies had included their final sample size achieved on the registry. The team did not attempt to contact the teams where the information was not available, due to time and resources available for this work; therefore, there may be a bias for those that have reported their sample size in the registry.

Sim and Lewis [12] recommend that an inflation factor is applied to the estimate of the standard deviation found in the pilot study. This is regardless of the size of the pilot study; however, the size of the inflation factor changes depending on the size of the PAFS that the information is based upon. Pilot studies with smaller sample sizes require larger inflation factors, therefore creating a larger definitive RCT. Researchers should not only be aware of applying this inflation factor, but that the inflation factor will change depending on the final achieved sample size (not the target sample size) in the pilot/feasibility study.

There were a number of limitations to note with this work. Due to the nature of the information available through the ISRCTN registry, some of the information such as condition and intervention type had many responses as “other”, this was the category supplied directly by the study team; however, it could be that they fit into a pre-existing category but the team wanted to add additional detail. This was not checked within this work and the study team response was used. Additionally, the number of studies in 2020 looks low compared with previous years; however, this could be due to a delayed retrospective registration rather than fewer studies. Further research is needed to update the data in the future to assess this properly. The ISRCTN registry only includes details on the total study sample size; therefore, the sample size per arm was calculated by taking this total and dividing it by the number of arms in the study assuming an equal ratio between the arms; however, this may not have always been the case.

The search terms used within the review of “pilot” and “feasibility” may have missed some eligible studies that had been described as phase 2 studies. To mitigate this, the search terms were completed on all records within the ISRCTN registration so that if any mention of pilot/feasibility was present this would be identified regardless of the title or study design terminology chosen. However, this does not completely remove the risk of missed eligible studies. This also impacts the generalisability of this review as those that are labelled phase 2 instead of pilot/feasibility may have different sample size characteristics. Additionally, as this review was limited to UK-based studies only, the results found here cannot be generalised internationally. Despite these limitations, we hope that this work provides an overview of a large number of PAFS using an online registry to understand the landscape of sample sizes.

Work published since the studies included in this review were designed has stated the need to look beyond setting a sample size for PAFS based on a singular consideration (such as precision of the standard deviation for example) and instead should consider all progression criteria which will ultimately determine the success of the pilot or feasibility study [23]. Future research could therefore consider whether the sample sizes currently recommended and being used are sufficient to achieve this.

## Conclusions

It has previously been stated that all RCTs need to justify the sample size they use, but a formal sample size calculation is not always required [8]. For PAFS, the sample size justification centres around having sufficient data to provide the key information needed to design a full RCT. There are numerous guidelines available to assist researchers in selecting this sample size for PAFS. This review suggests the target sample size used in practice is in line with the smaller recommendations but does not satisfy the most recent recommendations. Additionally, the findings are in line with designing a definitive RCT with a standardised effect size of less than 0.3, which was the median found in a review of definitive RCTs [22]. However, with less than half recruiting to target, these studies may not be providing suitable information in order to estimate key design features of definitive RCTs and researchers need to be aware of this when reporting results from PAFS and particularly when using these to design the definitive RCT.

### Supplementary Information

**Additional file 1. **

## Data Availability

The dataset supporting the conclusions of this article is available in the University of Sheffield’s ORDA repository, https://doi.org/10.15131/shef.data.22117745.v1.

## Appendix 1

Data extracted from the ISRCTN webpage (for definitions see https://www.isrctn.com/page/definitions) for each pilot/feasibility study was:

- 1. ISRCTN number
- 2. Study title
- 3. Country of recruitment
- 4. Funder and funding body type
- 5. Overall study start and end date
- 6. Overall study status
- 7. Date registered with ISRCTN
- 8. Primary and secondary study design
- 9. Condition category
- 10. Intervention type
- 11. Study type
- 12. Target number of participants
- 13.Total final enrolment

Further information was gathered manually from the information downloaded or the ISRCTN webpage itself (including any linked documentation if required for clarity):

- 1. Type (pilot/feasibility/both pilot and feasibility)
- 2. Number of treatment arms
- 3. Endpoint type (binary/continuous/time to event/categorical)
- 4. Funder (public/industry/charity/other)

## Appendix 2

**Table 5:** Median sample size per arm split by condition, study type and intervention type

	***N***	**Target sample size**		
		**Median (IQR)**	**Minimum**	**Maximum**
**Condition category**				
Cancer	72	30 (25, 46)	6	1598
Circulatory system	72	30 (20, 43)	4	200
Digestive system	21	25 (16, 30)	10	192
Ear, nose and throat	6	41 (19, 55)	15	71
Eye diseases	9	33 (20, 45)	10	50
Genetic diseases	3	25 (20, 29)	15	32
Haematological disorders	5	25 (20, 28)	20	65
Infections and infestations	24	50 (35, 88)	10	350
Injury, occupational diseases, poisoning	24	25 (15, 33)	10	51
Mental and behavioural disorders	187	30 (22, 40)	5	602
Musculoskeletal diseases	55	26 (24, 40)	10	232
Neonatal diseases	4	57 (35, 87)	20	125
Nervous system diseases	46	25 (16, 39)	5	383
Nutritional, metabolic, endocrine	55	30 (20, 49)	10	650
Oral health	15	36 (20, 65)	10	400
Pregnancy and childbirth	27	45 (30, 70)	5	175
Respiratory	37	30 (25, 40)	15	100
Signs and symptoms	15	30 (20, 42)	5	84
Skin and connective tissue diseases	9	35 (25, 40)	10	60
Surgery	15	35 (25, 43)	15	58
Urological and genital diseases	28	25 (18, 52)	6	400
Unknown	32	46 (30, 76)	12	350
**Study type**				
Diagnostic	8	63 (41, 79)	6	125
Prevention	63	40 (29, 70)	10	650
Quality of life	67	30 (21, 50)	8	350
Screening	12	60 (40, 245)	20	1598
Treatment	568	30 (20, 42)	4	602
Other^a^	43	40 (25, 56)	6	140
**Intervention type**				
Behavioural	231	30 (20, 46)	6	650
Biological/vaccine	4	17 (14, 27)	10	50
Device	43	25 (17, 37)	5	200
Drug	50	30 (20, 57)	12	265
Mixed	24	29 (20, 60)	10	100
Procedure/surgery	64	25 (20, 37)	4	232
Supplement	18	25 (20, 32)	15	100
Other^a^	325	30 (24, 50)	5	1598
Unknown	2	23 (19, 26)	15	30

^a^Other defined by the study team in the ISRCTN web registry

## Abbreviations

ICMJE: International Committee of Medical Journal Editors
IQR: Interquartile range
ISRCTN: International Standard Randomised Controlled Trial Number
MRC: Medical Research Council
NHS: National Health Service
NIHR: National Institute for Health and Care Research
PAFS: Pilot and Feasibility Studies
WHO: World Health Organization
